# Stiffness optimization and reliable design of a hip implant by using the potential of additive manufacturing processes

**DOI:** 10.1186/s12938-022-00990-z

**Published:** 2022-04-02

**Authors:** Lena Risse, Steven Woodcock, Jan-Peter Brüggemann, Gunter Kullmer, Hans Albert Richard

**Affiliations:** 1 Institute of Applied Mechanics, Paderborn University, Pohlweg 47-49, 33098 Paderborn, Germany; 2 Direct Manufacturing Research Center, Paderborn University, Pohlweg 47-49, 33098 Paderborn, Germany; 3 Advanced Mechanical Engineering GmbH, Carlo-Schmid-Allee 3, 44263 Dortmund, Germany

**Keywords:** Structural optimization, Hip implant, Selective laser melting

## Abstract

**Background:**

Due to the steadily increasing life expectancy of the population, the need for medical aids to maintain the previous quality of life is growing. The basis for independent mobility is a functional locomotor system. The hip joint can be so badly damaged by everyday wear or accelerated by illness that reconstruction by means of endoprostheses is necessary.

**Results:**

In order to ensure a high quality of life for the patient after this procedure as well as a long service life of the prosthesis, a high-quality design is required, so that many different aspects have to be taken into account when developing prostheses. Long-term medical studies show that the service life and operational safety of a hip prosthesis by best possible adaptation of the stiffness to that of the bone can be increased. The use of additive manufacturing processes enables to specifically change the stiffness of implant structures.

**Conclusions:**

Reduced implant stiffness leads to an increase in stress in the surrounding bone and thus to a reduction in bone resorption. Numerical methods are used to demonstrate this fact in the hip implant developed. The safety of use is nevertheless ensured by evaluating and taking into account the stresses that occur for critical load cases. These results are a promising basis to enable longer service life of prostheses in the future.

## Introduction

In Germany, approximately 210,000 initial implantations of total hip endoprostheses and 30,000 revision operations were carried out in 2011 [1]. In addition, about 125,000 knee prostheses were implanted annually [2]. Between 2007 and 2017, the number of implants increased by 30–40% [3]. This makes this surgical procedure one of the most common orthopedic treatments of our time [4]. The aim of the surgery is to improve the patient's quality of life by restoring freedom of movement in the affected joint and reducing pain [5]. Continuous research in the field of hip endoprosthetics has led to innovations in technology, materials science, surgical techniques and methods of fixation and sterilization, which have contributed to increasing the life span of implants. Today, 75% of implanted hip endoprostheses can remain in the body for up to 15 years [6, 7].

Despite the constant innovations, aseptic loosening of the prosthesis, caused by so-called "stress shielding", remains an existing problem [7, 8]. Because the prosthesis is much more rigid than the bone, there is a lack of stress in the contact zone between the prosthesis and the femur, so that the bone in the area of the prosthesis recedes [7]. Furthermore, the aforementioned difference in stiffness can lead to pain for the patient. Numerous attempts have been made to reduce the stiffness and eliminate the associated complications [9]. This can result in more complex geometries, such as lattice structures, so that modern manufacturing methods, like the selective laser melting (SLM) process [10], offer themselves for promising further research.

In the context of this paper, the high design freedom of additive manufacturing processes in combination with computer aided engineering (CAE) methods is used to provide approaches to solve the existing stiffness problem in hip endoprosthetics. Using stress-adapted geometries and the finite element method, stiffness-adapted variants of a short shaft hip endoprosthesis are developed in an iterative process. Further optimization steps are continuously derived from the analysis of stresses and deformations in prosthesis and bone. The optimization goal is the reduction of the current stiffness and the resulting increase and homogenization of the stress in the surrounding bone. Furthermore, the focus is on improved fixation and durability with regard to the period of use as well as a compact, bone-saving design and direct force transmission.

## Results

**Fig. 1:**
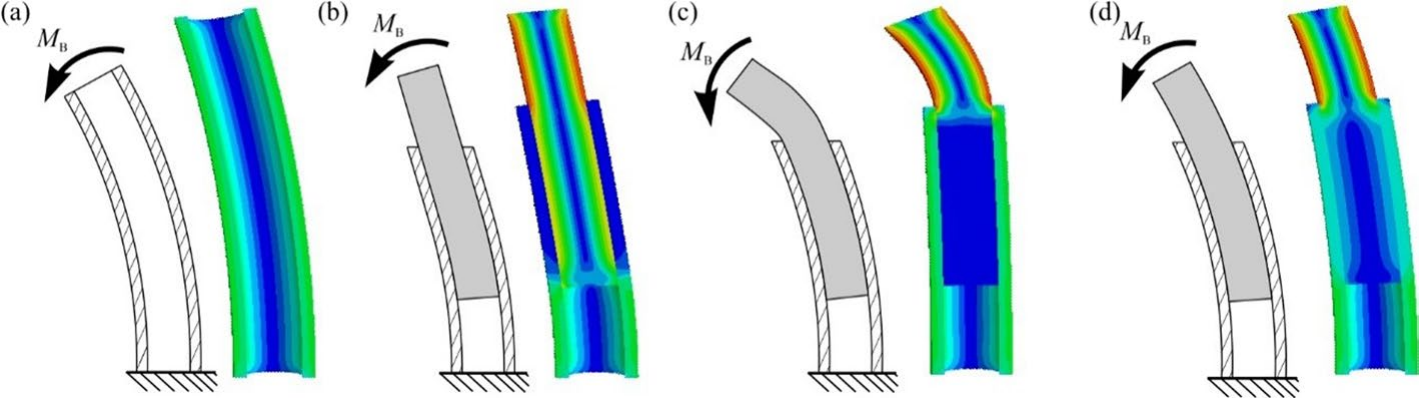
Qualitative numerical analysis to illustrate the influence of implant stiffness on the stress situation in the bone. (**a**) Healthy bone without implant. (**b**) Bone with too stiff implant ("stress shielding"). (**c**) Bone with implant that is too flexible. (**d**) Implant with adjusted stiffness

The relevance of adapting the implant stiffness to the possible time of implant use is visualized in Fig. 1. Implants of different stiffness placed in a schematic bone are subjected to bending loads and the resulting qualitative stress is compared to that of the healthy bone (Fig. 1a). In this context, blue areas reflect low stress and red areas high stress. Figure 1b illustrates the stress situation in the bone when using an implant that is too stiff. The entire load is carried by the implant, so that increased stress in the bone occurs only in the distal end, while the proximal part is free of stress. This effect, known as "stress shielding", leads to loosening of the prosthesis, since the unstressed bone is gradually degraded. An implant that is too flexible does not take up enough of the applied load, so that the entire bone is subjected to greater stress (Fig. 1c). An implant with a stiffness adapted to the bone has only a minor effect on the loading situation compared to healthy bone (Fig. 1d). Both the bone and the implant are stressed over the entire implant length. This leads to a good growth of the implant and avoids loosening.

### Optimization results

**Fig. 2:**
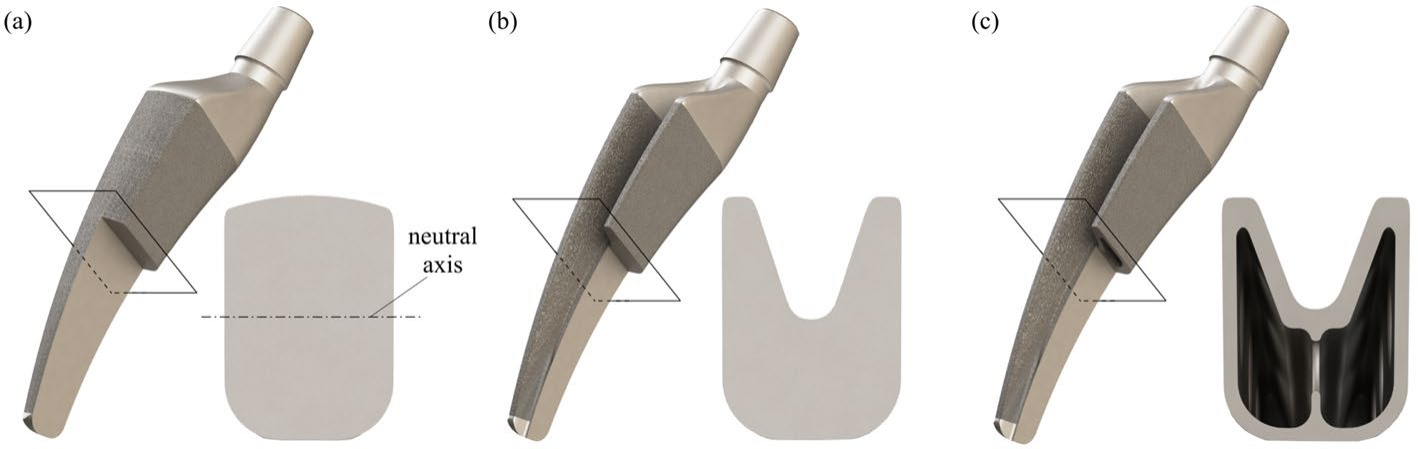
Geometry adaptation for stiffness reduction. (**a**) Initial model (full rectangular profile, *I*_max_ = 4915 mm^4^, *I*_min_ = 2507 mm^4^) in partial section. (**b**) Optimized prosthesis with U-profile (*I*_max_ = 2953 mm^4^, *I*_min_ = 2276 mm^4^) as basic geometry in partial section. (**c**) Prosthesis with U-hollow profile (*I*_max_ = 1387 mm^4^, *I*_min_ = 1043 mm^4^) in partial section

The optimization process is carried out by systematically changing the cross-sectional profile of the hip prosthesis for stepwise stiffness adjustment, which is shown in Fig. 2. Since the two load cases mainly cause bending stress, the moment of inertia *I*_y_ of the surface is decisive for the stiffness of the implant. The more material is placed far away from the neutral axis, the higher is the resulting moment of inertia. This can be illustrated using the formula for rectangular profiles:

1 $$ I_{y} = \frac{{b \cdot h^{3} }}{12}, $$

where *b* is the width of the profile parallel to the neutral axis and *h* is the height of the profile perpendicular to the neutral axis.

The initial model is similar to the standard cross-section of commercially available short shaft hip endoprostheses. The nearly rectangular cross-section (Fig. 2a) has the highest moment of inertia of the three variants. A first reduction of the moment of inertia is achieved by changing the cross-section. Compared to the initial model, the U-profile has less material far away from the neutral layer (Fig. 2b).

Since the size of the outer cross-section should not be reduced to ensure adequate anchoring in the bone, material inside the implant is removed to further reduce stiffness. The final basic geometry, designed as a U-profile with hollow chambers, is visualized in Fig. 2c. Since the shaft area of the prosthesis is to be considered in connection with the bone after implantation, the reduced torsional stiffness of the prosthesis due to the change in geometry is negligible. The changed cross-sectional geometry also provides a higher torsional stiffness, which enables a more solid anchorage in the femur.

### Final design using numerical methods

**Fig. 3:**
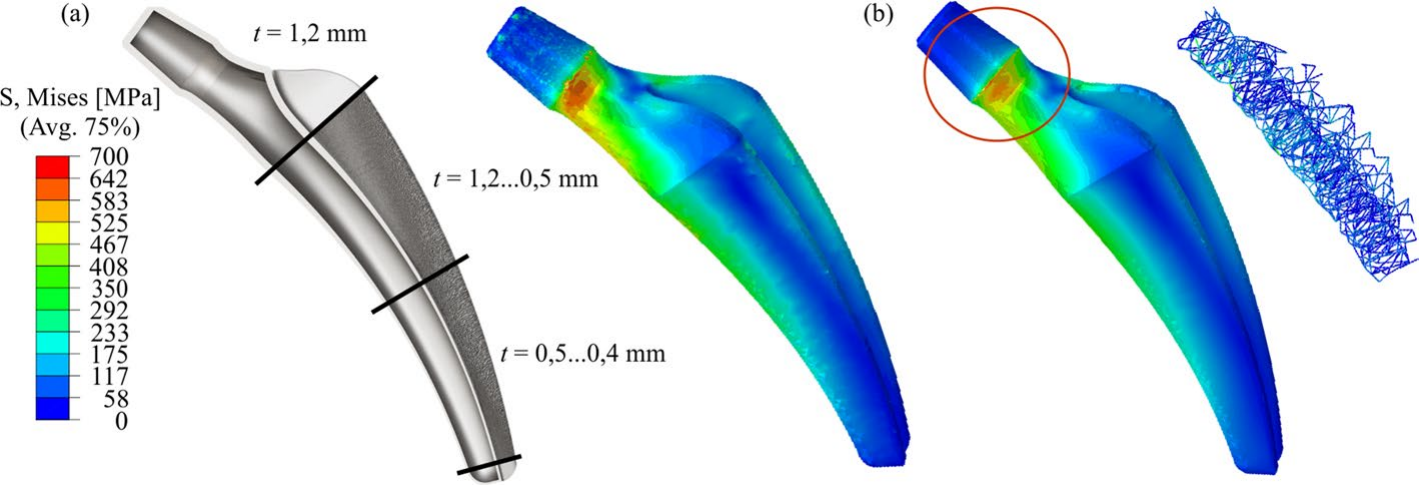
Development of the final prosthesis variant under consideration of SLM-process-related restrictions. (**a**) Determination of a suitable wall thickness dimensioning. (**b**) Use of grid structures for local load-bearing capacity increase. The red circle indicates the region with reduced stresses because of the inner grid structure

The choice of a U-hollow profile with constant wall thickness is not appropriate with the aim of achieving the most homogeneous material utilization possible. Therefore, a variation of wall thickness is carried out in an iterative process. Numerical analyses are continuously used to check that the design is safe for use. In the shaft area, the wall thicknesses can be reduced so that the desired reduction in stiffness is achieved at the same time. In the neck area of the prosthesis, which is not implanted in the bone, a high degree of stiffness and load-bearing capacity is required to ensure that the function is fulfilled. Therefore, the wall thickness in the neck area is thicker. Finally, the stress-adjusted wall thickness dimensioning is shown in Fig. 3a results. Production-related restrictions in the SLM process prevent further reduction of the wall thickness in the distal shaft area. The numerical analysis for the critical load case stumbling illustrates compliance with the maximum permissible stress *σ*_zul_ of the prosthesis.

The optimization task can be considered in two parts. In the implanted stem area of the prosthesis, stiffness reduction is the primary goal, while in the more highly stressed neck area, the focus is on sufficiently high strength. In the highly loaded neck area of the prosthesis, a local stress increase is visible. To homogenize the stresses and to increase the load capacity, a grid structure is inserted locally in the high-stressed areas (Fig. 3b). By this procedure, the stress in the neck area can be reduced without significantly influencing the stiffness of the shaft and the prosthesis weight.

**Fig. 4:**
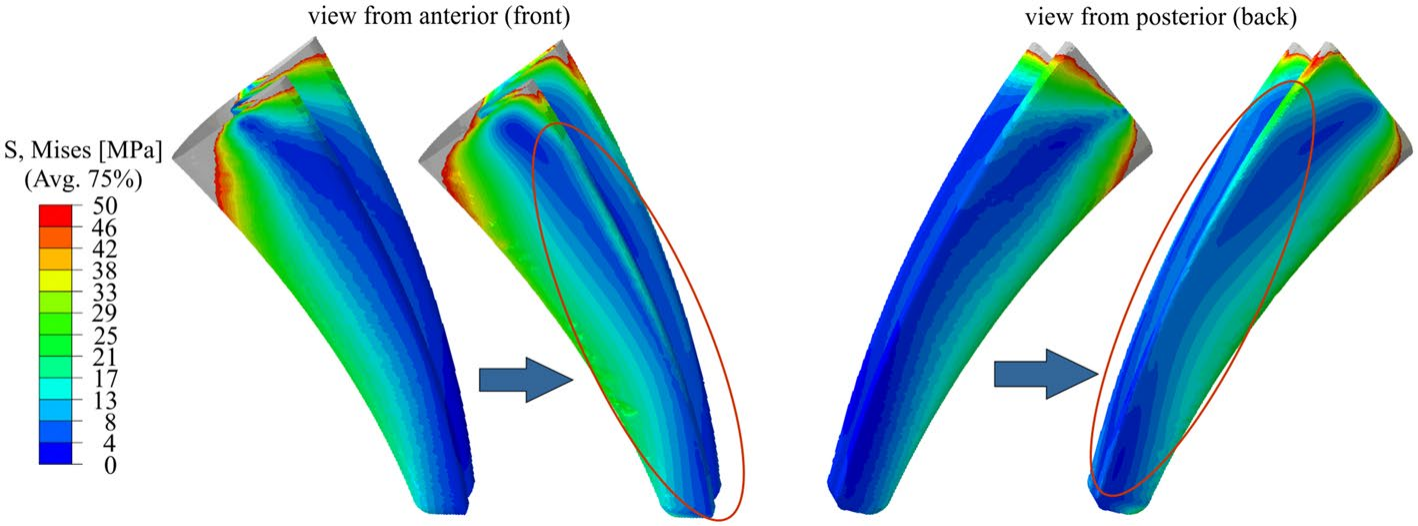
Analysis of the change in the stress situation within the contact surface of the femur due to the stiffness adjustment. The red ellipses indicate the regions with increased stresses

The optimization measures carried out result in a reduction of the stiffness in the shaft area of the prosthesis. Furthermore, the local use of grid structures in the highly stressed neck area of the implant has increased its load-bearing capacity and reduced the resulting stresses. To validate the success of the stiffness reduction in the shaft area of the prosthesis, the loading situations within the contact surface of the femur at the beginning of the optimization process and at the end are compared for the load case stumbling in Fig. 4. Successful structural optimization leads to an increase in the stress on the bone tissue surrounding the prosthesis.

The reduction of the stress-free and low-stressed areas becomes visible in the view from anterior (front) as well as from posterior (back). The result is a more homogeneously stressed bone contact surface, which allows a more extensive transfer of stress to the bone and reduces the risk of bone degradation due to stress shielding.

### Experimental testing

**Fig. 5:**
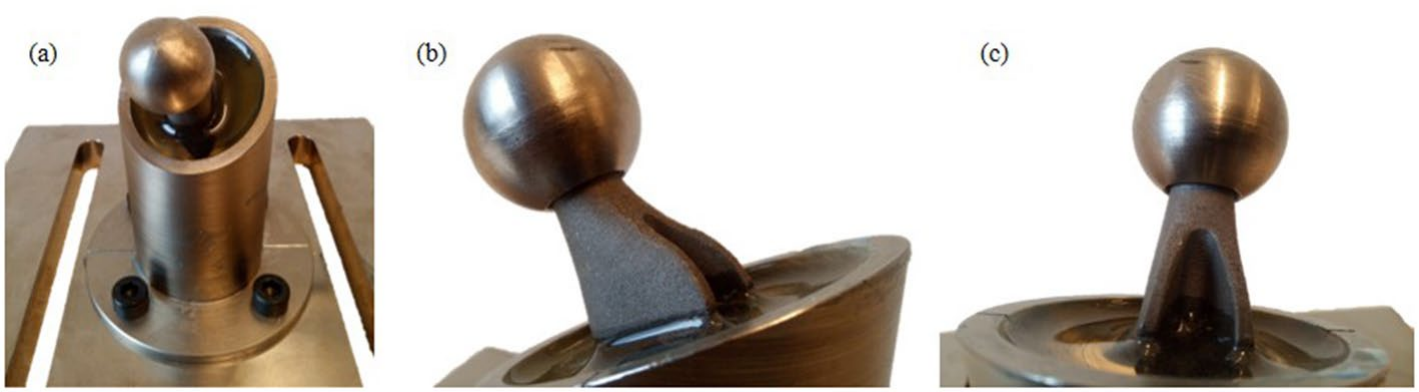
Experimentally tested prototype. (**a**) Prosthesis embedded in resin. (**b**) Detailed view of the undamaged neck of the prosthesis after experimental testing. (**c**) Detailed view of the undamaged neck of the prosthesis after experimental testing from a different perspective

The short shaft hip endoprostheses are built up "standing" in order to keep the process-induced internal stresses in the component as small as possible in the case of the titanium aluminum alloy due to the smaller surface area to be exposed. In addition, the proportion of support structure is minimized in this way. In order to validate the design assumptions and verify the operational safety of the prosthesis, experimental tests were performed following the applicable testing standards. The experimental tests have been passed successfully. No visible deformation, no deformation affecting the test process or visible cracks occurred (see Fig. 5).

The numerical design of the prototype developed can be described as reliable. This was also confirmed by experimental investigations. However, various questions still need to be clarified before it can actually be used in the human body. For example, powder removal, behavior in contact with human tissue and various further investigations are necessary in this context. Nevertheless, these results show promising potential for the use of selective laser melting to reduce the difference in stiffness between bone and implants and thus to reduce stress shielding and aseptic loosening.

## Discussion

Many factors are relevant when it comes to the design and approval of novel implants. Despite the enormous effort involved, continuous further development is necessary to meet the requirements of an aging society. Additive manufacturing processes are a promising element in this further development. Thanks to the great freedom of design, it is possible to tailor geometries more closely to the actual application, so that the problem of stress shielding, among other things, can be effectively countered.

Other studies have already exploited the possibilities of the SLM process for prosthetics [11, 12]. In this way, for example, porous structures could be created to promote the ingrowth of bone into the prosthetic structure. In the approach chosen here, this was not done because it makes the prosthesis more difficult to remove and revise, and young patients were chosen as the target group. The main aim of these investigations is to enable stiffness adjustment and the associated increase in stresses in the surrounding bone.

The finite element method is a suitable tool to be able to investigate the mechanical effects of the changed prosthesis geometry. A variety of previous investigations [13–15] provide access to almost realistic simulation boundary conditions. In a study by Cilla et al. [16], a FE model with a complete femur including all joint and muscle forces is used to investigate the effects of prosthesis stem modifications to reduce stress shielding. The FE model used in this study (see Fig. 5) considers the femur above the knee joint and the joint and muscle forces applied at the proximal end. Although the model used here is not as sophisticated as the model used by Cilla, it is suitable for investigating the principal effects of stem modifications on the stresses on the bone in the contact area. Nevertheless, due to the high safety relevance, both extensive experimental and clinical studies are necessary to validate the results.

The change in cross-section to a U-profile represents a new approach that should bring various advantages. On the one hand, the U-shape allows the reduction of the moment of inertia without the implantation area becoming too small. On the other hand, twisting of the implant after implantation is prevented and, in addition, a larger contact area between the prosthesis and the bone is created so that a better adhesion can be realized. Due to the cross-sectional size tapering in the distal direction, sinking of the prosthesis stem into the bone shaft is prevented. In addition, the increased contact surface between bone and implant results in a higher connection strength.

In order to be able to verify the actual influence of the new implant geometry with adapted stiffness on the service life of the implant and the reduction of bone resorption, far-reaching clinical studies are necessary. However, the results of the numerical investigations are promising and the safety of use has already been confirmed experimentally. However, the experimental investigations only represent a kind of initial feasibility study. The basic operational reliability with regard to the assumed mechanical loads could be confirmed. The special conditions of use within the human body and other essential test criteria have not yet been investigated and evaluated as part of this study. Therefore, among other things further investigations are required to remove possible powder residues before use in living tissue.

## Conclusion

Within the scope of this article, a stiffness-adapted short shaft hip endoprosthesis could be developed by targeted use of the potentials of selective laser melting, in particular the possibility of creating filigree internal grid structures and variable wall thicknesses as well as internal cavities. By numerical analysis of the stress situations of bone and implant, the problem of "stress shielding" and thus potential problems of the patient could be reduced and the expected service life of the prosthesis increased. The stiffness-adjusted hip endoprostheses were checked for their operational reliability by numerical methods. The design was validated by experimental component tests according to the ISO testing standards.

The findings on stiffness adjustment by exploiting the potential of selective laser melting can now be transferred to other components. Especially for implants, the problem of the stiffness difference between bone and implant is of immense importance, but also technical applications can profit from these considerations.

## Methods

### Preliminary considerations

In order to be able to carry out a systematic optimization process, some preliminary considerations are necessary. These concern on the one hand the desired requirements for the implant and the analysis of various factors influencing the duration of use, and on the other hand the derivation of further optimization steps on the basis of previous preliminary studies.

The best possible observance and retention of the biomechanics in the hip joint and thus the avoidance of major impairments is a primary goal of artificial joint replacement. With its structure, the hip ensures the biomechanical function of enabling movements between the pelvis and the femur and at the same time ensuring the transmission of forces [17]. In order to achieve a good freedom of movement of the joint, the diameter of the femoral neck is smaller than that of the femoral head. Further mechanical parameters are influenced by the centrum–collum–diaphyseal angle (CCD angle). Depending on the angle, the loads acting on the hip joint change. Normally the CCD angle is 125° [17].

The femur is the largest bone in the human body and belongs to the group of long bones [18]. The tube-like bone shaft is made of a solid substance, the compacta. The bone ends consist of a spongy structure, the cancellous bone [18, 19]. The bone structure is always in continuous remodeling, so that optimal force absorption is guaranteed at all times. Less stressed bone material is reduced, while more stressed areas are strengthened [20, 21].

The hip joint is exposed to a wide range of stress situations in everyday life. When designing an artificial hip joint replacement, these load situations must be quantified in order to guarantee the safety of the implant. The load assumptions used are based on a study by Bergmann et al. [14]. In this context, a prosthesis stem was developed for data acquisition, which was equipped with appropriate measuring technology, including telemetric data transmission [14]. Within the scope of this article, two exemplary load cases for the development process are taken from this study. On the one hand, walking is considered as an everyday load on the hip joint for the design against failure due to fatigue. The contact force *F*_K_ between the *caput femoris* (femoral head) and the *acetabulum* is 280% of body weight. On the other hand, the stumbling that causes the highest stress (870% of body weight) is used to exclude a forced fracture [14]. To determine the angle of force application *α*, the one-legged stance is used as a basis, since the stress on the hip joint is highest when only one leg is loaded. In this case, the resulting angle of force application *α* to the vertical is 16° [22]. The prosthesis should be designed for a middle-aged male patient (weight 79 kg). Accordingly, the contact force *F*_K,walking_ = 2 170 N occurs during walking. When stumbling, forces of *F*_K,stumbling_ = 6 742 N act [23].

Three factors have a major influence on the stability of an implant: the fit, the fixation and the stiffness. With regard to stability, the primary stability immediately after implantation and the stability after growth must be considered. Poor primary stability leads to micromovements of the prosthesis, resulting in pain for the patient. Poor stability after growth can result from bone resorption caused by inadequate load transfer to the bone [24].

A high fit (form fit between prosthesis and implant) has a positive effect on the primary stability, but a negative effect on the stability after growth. Accordingly, a suitable compromise must be chosen in this context. With regard to fixation in the femur, there are two variants: anchoring with bone cement and cementless anchoring. For younger patients, the cementless version is usually preferred due to numerous advantages, such as easier revision surgery and the avoidance of tissue damage caused by the cement polymer [25–28]. Fixation with bone cement has a positive effect on primary stability, but loosening symptoms may occur over time. The reduction of the stiffness to a value similar to that of the bone has positive effects on the primary and long-term stability of the hip implant.

One way to vary the stiffness of the implant is the choice of the material. It must be ensured that the selected material not only provides the desired stiffness, but also guarantees the fulfilment of the function by sufficiently good mechanical characteristics. Furthermore, it has to be biocompatible. In this way, damage to the surrounding tissue due to sufficient chemical and biological compatibility of implant and body is excluded [29]. A material that meets the above-mentioned requirements and represents an alternative from a stiffness point of view is the titanium aluminum alloy Ti6-4, which can be processed reliably by selective laser melting. An overview of the mechanical material characteristics, determined on laser-melted test specimens, is shown in Table 1.

**Table 1:** Material characteristics of the Ti6-4 alloy [30]

Material	Yield strength [MPa]	Tensile strength [MPa]	Elongation at break [%]	Young's modulus [MPa]
Ti6-4	912	1 005	8.3	115 000

A striking feature is the low Young’s modulus (half the Young’s modulus of steel) compared to other biocompatible metallic materials in combination with high strength values, which has a positive influence on the stiffness optimization of implants. Another advantageous aspect of this material is its good osteogenetic property. Since Ti6-4 is bioinert, no harmful interactions with the body's own tissue occur.

In order to guarantee the safety of the implant, a strength and fatigue strength test is carried out. The basic statement of these two verifications is that the effective stresses in the component must always be less than the load-bearing capacity of the material [31]. For the verification that no failure due to plastic deformation occurs, von Mises equivalent stress is used for the load case stumbling. To determine the permissible stress on the material side, the yield strength is divided by a safety factor *S*_F_ against plastic deformation [31].

Cyclic loads usually cause failure by fatigue crack growth. For this reason, when designing the prosthesis for the load case walking, the stresses are evaluated using the equivalent stress according to Navier, since cracks always grow perpendicular to the maximum principal stress [31]. The allowable stress is calculated taking into account the fatigue strength of the material, the technological size coefficient, the surface roughness and a safety factor.

### Boundary conditions for numerical simulation

In order to achieve a typical average FE model for the femur that is as close to reality as possible CT data of the femur of different 40- to 60-year-old male patients are taken and transferred to 3D-volume models. The slice thickness and the cross-sectional resolution were isotropic and less than 1 mm in all CT images in each case. Using Materialise Mimics, CT data of different femurs were analyzed in terms of their density distribution and thus in terms of Young's modulus. The Young’s modulus of the femur is assumed to be variable in order to best reflect the prevailing properties of the cortical and cancellous bone. Based on literature values and the results of the density distribution of various CT examinations, areas were defined in each case to which a specific Young's modulus was assigned. The cortical area is assigned a Young's modulus of 20 GPa, filled with bone marrow (1 MPa). In a transitional area between the cortical and cancellous bone, the Young's modulus gradually decreases until it varies between 100 and 2000 MPa in the cancellous head. However, different areas with different linear isotropic material properties are assumed to reduce the computing time while nevertheless realistic stiffness distributions were present. For the numerical simulations with the software Abaqus CAE 2017. The inner lattice structure was meshed with beam elements, all other components and prosthetic areas were meshed with quadratic tetrahedral elements (C3D10). The maximum size of the tetrahedral elements is defined as 1.5 mm. Cross-section transitions, notches and other areas with a high stress gradient are meshed correspondingly finer to avoid unwanted numerical errors. Due to the small deformations a geometrical linear calculation was carried out.

**Fig. 6:**
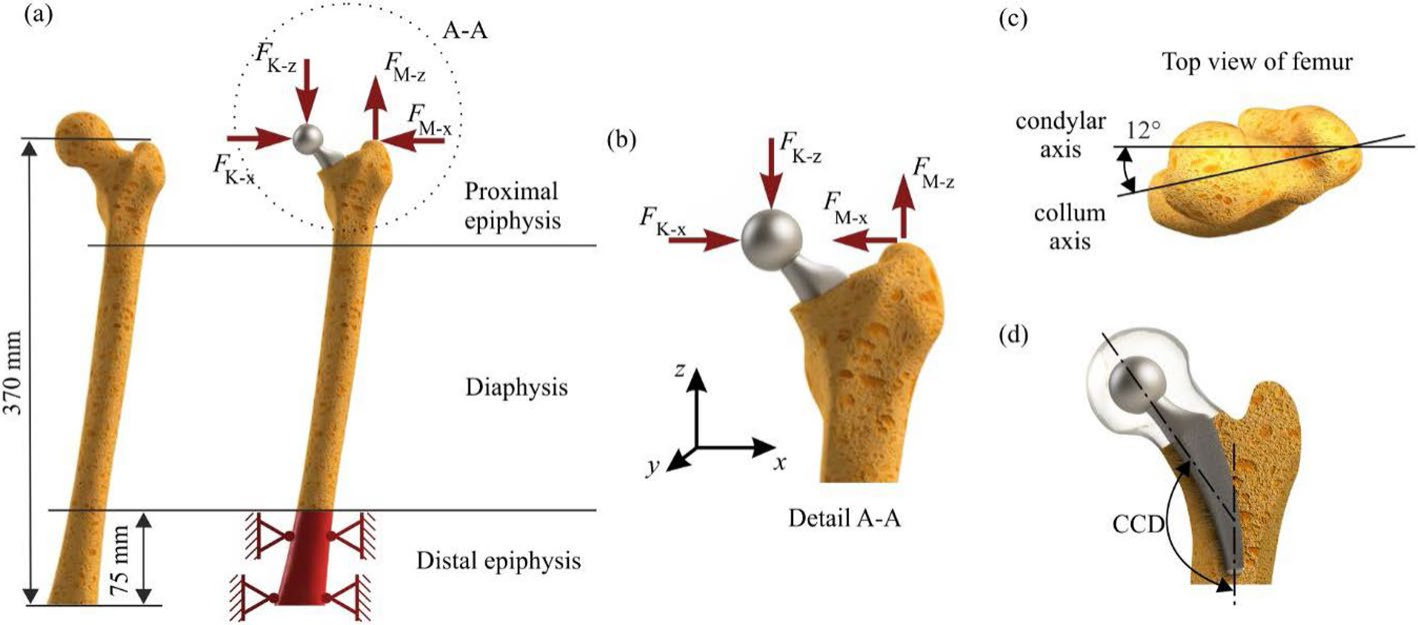
Boundary conditions and influencing factors for numerical analysis. (**a**) Boundary and constraint conditions for the FE model. (**b**) Details of the load application points A-A. (**c**) Alignment of the femur according to the anatomical axes. (**d**) Relevance of CCD angle

The femoral stump used for the simulation is clamped firmly in the anatomically correct position at its end (Fig. 6a). Its position is tilted by 9° in lateral direction. Furthermore, the collum axis of the femoral head is rotated 12° in an anterior direction with respect to the condylar axis of the distal femur (Fig. 6c) and is described by the antetorsion angle [32]. In the Finite Element (FE) study, the situation after complete healing and attachment of the bone to the prosthesis is considered. The contact situation of prosthesis and surrounding bone is therefore modeled as tie-constraint. Furthermore, the aim is to find measures to modify the shaft of the prosthesis to avoid stress shielding on the bone contact surface. Thus, regions with too low stresses on the bone contact surface are unwanted. Preliminary studies with different frictional coefficients ranging from 0.1 to 0.8 showed that the stresses on the bone surface decrease slightly with increasing frictional coefficient. Since higher stresses are wanted and tie contact is the upper limit of frictional contact, tie contact represents the worst case to reach the aim. Also for this reason tie contact is chosen to check that with the selected measures to alter the shaft of the prosthesis even the lowest possible stresses on the bone surface are high enough to avoid stress shielding.

The respective joint contact forces in *x*- and *z*-direction (*F*_K-*x*_, *F*_K-*z*_) are applied as distributed forces in the contact area (923 mm^2^) via the ball head of the prosthesis. An additional load is added by the abductor muscle group (*F*_M-*x*_, *F*_M-*z*_) (Fig. 6b). Gluteus abductors reduce the extension load in the proximal part of the femoral neck to such an extent that there are effects on the subsequent design of a prosthesis in terms of its stiffness [33, 34]. The amount of muscle force applied is 1.1 times the body weight [35]. To take into account the natural anatomical structure, various dimensions as well as the geometric shape of the prosthesis are relevant; see Fig. 6.

The prosthesis is intended to replicate the healthy femur as closely as possible, for example, compliance with the existing CCD angle is relevant (Fig. 6d). The basic geometry developed in the context of this article can be adapted to any variation of the CCD angle. Further relevant dimensions are the head and neck diameter of the prosthesis as well as the cone dimensions. They are chosen with regard to [10] to ensure the patient's freedom of movement, a sufficient joint stability and a permanent and stable fit of the connection between ball head and prosthesis.

The optimization of the prosthesis geometry is carried out with the aid of CAD and FEM software. No optimization software is used; instead, knowledge of technical mechanics, structural mechanics and biomechanics is incorporated into the manual optimization process.

### Manufacturing and experimental testing

For the production of optimized hip endoprostheses, a process that offers a high degree of design freedom is required. Selective laser melting is chosen as it empowers the production of both filigree, internal and complex geometries [36]. Thus, almost no geometric restrictions have to be taken into account for the optimization process and the optimization success is not impaired by the choice of the manufacturing process. The selected material Ti6-4 can be processed reliably on the SLM280 2.0 machine with standard parameters for this material and is approved for the production of implants. Since the adhesion of bone is enhanced by microporosities on the surfaces of implants, the choice of the SLM process can additionally be seen as positive. The non-implanted neck and head area of the prosthesis can be polished after fabrication to ensure improved fatigue properties as it increases the surface condition coefficient. Since not perfectly isotropic material properties result from the SLM process, the material parameters for the design direction with the lowest mechanical properties were selected for determining the maximum allowable stresses in the prosthesis and nevertheless isotropic material is assumed in the FE simulation.

Experimental investigations are carried out to validate the numerically determined operational reliability. The operational reliability of the implant is determined numerically beforehand. For this purpose, the maximum permissible stresses are determined in advance by means of a fatigue strength verification. In addition to a conservative safety factor, a conservative estimate of the surface quality and the corresponding reduction factor are used to plan a further safety reserve. Thus, a fatigue-resistant design should be ensured and no damage or plastic deformation should occur during use.

First of all, the selected load assumptions based on real measurements are used for the experimental tests. International standards have been published in order to guarantee a standardized testing of this medical product. ISO 7206: Implants for surgery—Partial and total hip joint prostheses describes in a total of ten documents the requirements for experimental tests of hip prostheses, which they must pass before the start of a clinical study.

Part 4: Determination of endurance properties and performance of stemmed femoral components [37] and Part 6: Endurance properties testing and performance requirements of neck region of stemmed femoral components [38] are particularly relevant for testing the optimized short stem hip endoprosthesis.

Based on the specifications from the ISO standards, two devices for the experimental testing of the optimized hip endoprosthesis are being developed, which are compatible with the available testing machine. This is an in-house development by the Institute of Applied Mechanics for carrying out experimental investigations on various additively manufactured components. With the aid of a linear motor, static and cyclic test forces can be applied and the component behavior recorded The prosthesis is embedded in an epoxy resin for the cyclic tests. The positioning and embedding depth of the prosthesis for the experimental investigations were ensured with the aid of an embedding device. The required boundary conditions for the experimental test are also clearly defined in ISO7206-4 and ISO 7206–6 and were replicated as accurately as possible for these experimental studies.

## Data Availability

The datasets generated during and/or analyzed during the current study available from the corresponding author on reasonable request.

## Abbreviations

CAD: Computer aided design
CAE: Computer aided engineering
CCD: Centrum–collum–diaphyseal
CT: Computer tomography
FEM: Finite element method
SLM: Selective laser melting
